# Supplementary material for: Reversible polymer-gel transition for ultra-stretchable chip-integrated circuits through self-soldering and self-coating and self-healing
Source: Nat Commun. 2021 Aug 3;12:4666. doi: 10.1038/s41467-021-25008-5 (PMC8333313; doi:10.1038/s41467-021-25008-5)
Supplement: Supplementary file 1 — Supplementary Information [file 41467_2021_25008_MOESM1_ESM.pdf]

Supplementary Information

Reversible Polymer-Gel transition for Ultra-Stretchable Chip-Integrated Circuits through Self-Soldering and Self-Coating and Self-Healing

Pedro Alhais Lopes[1], Bruno Santos[1], Anibal T. de Almeida[1], Mahmoud Tavakoli[1*]

[1]Soft and Printed Microelectronics Lab
University of Coimbra
Coimbra, Portugal
[*]e-mail: mahmoud@isr.uc.pt

[*]Corresponding Author

Supplementary Figures

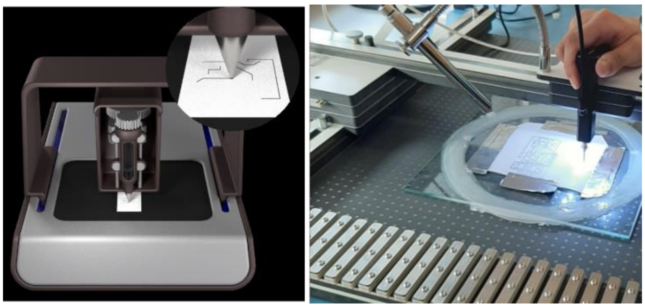

Supplementary Figure 1. **Printing and the pick and place setup**. Voltera V-One was used to print the AgInGa ink. Chips are placed on the circuit using a pick and place machine (eC-placer Eurocircuits).

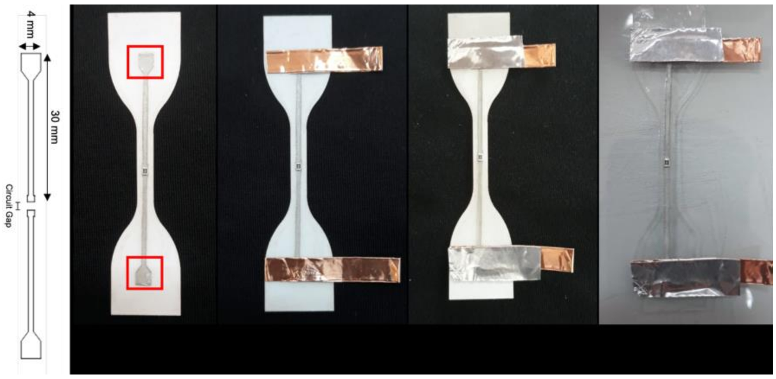

Supplementary Figure 2. **Dogbone geometry**. Dimensions, and examples of the prepared samples.

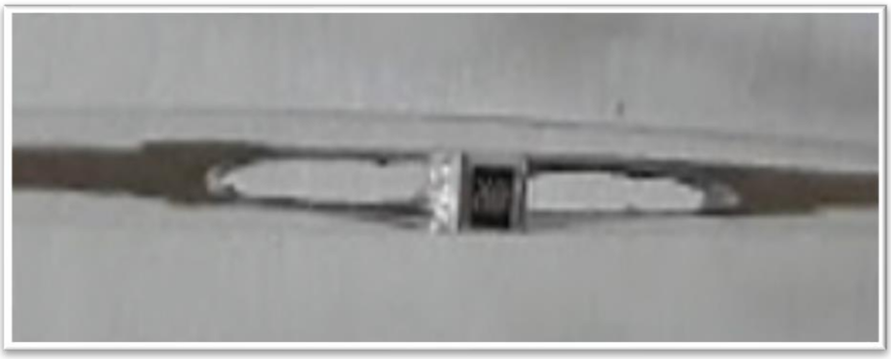

Supplementary Figure 3. Sample under strain. Moment captured right before the failure. At this state, the electrical interface is still functional, i.e. the mechanical failure occurs prior to the electrical failure. This visible holes always start on the interface between the microchip and the printed traces.

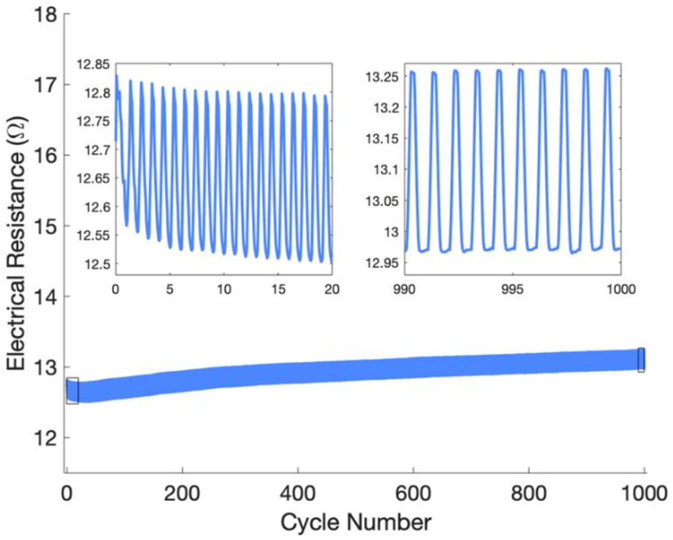

Supplementary Figure 4. Electromechanical characterization. Electromechanical coupling of a printed sample with a 10Ω resistor, under repetitive 40% strain.

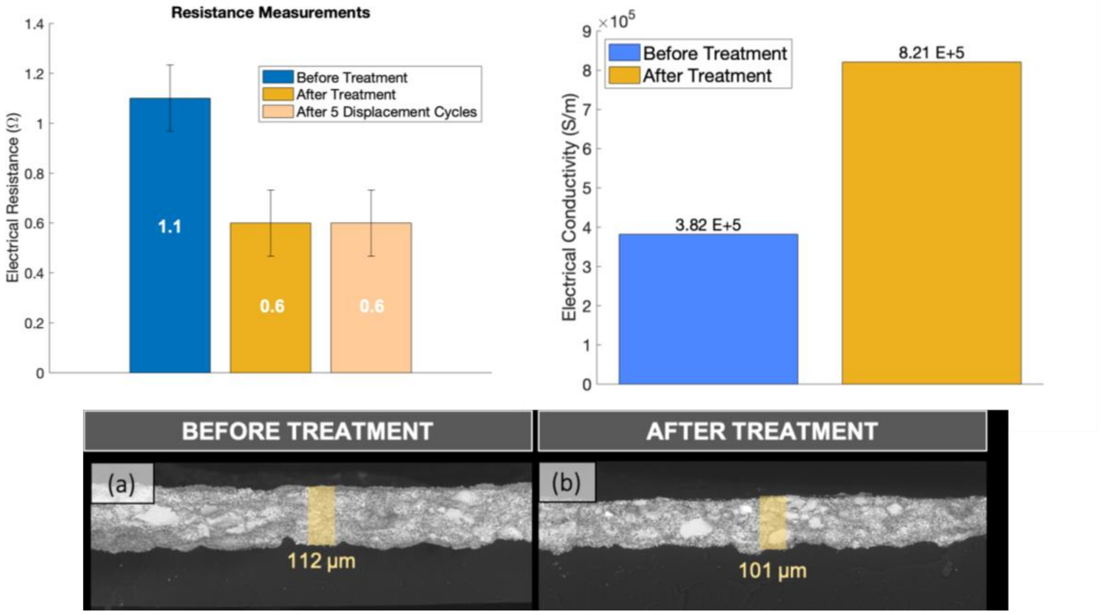

Supplementary Figure 5. **Characterization of the printed samples.** Characterization before and after the vapor exposure. Before Treatment: Thickness = 112 µm; Electrical Resistance Average= 1.1Ω. After Treatment: Thickness = 101 µm; Electrical Resistance average = 0.6Ω.

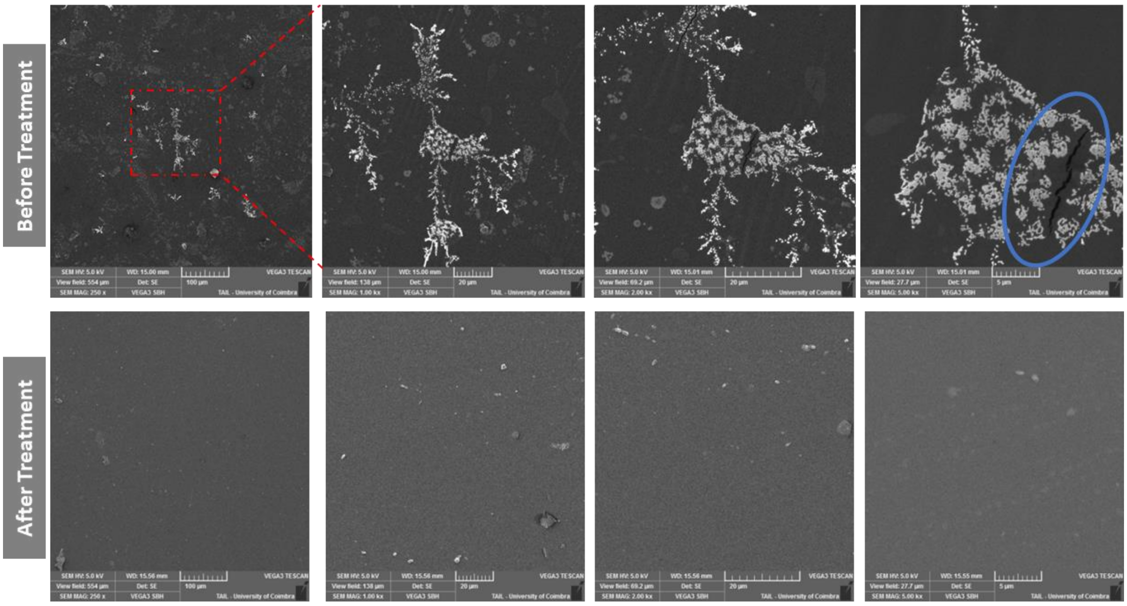

Supplementary Figure 6. **Scanning Electron Microscopy**. A SIS only sample before and after the vapor exposure. SEM images with various magnification. After the vapor exposure, the surface of the substrate is significantly smoother, cracks are healed, and undissolved SIS powder is eliminated.

Weight %

	C	Ga	Ag	In
**Z1**	1	2	32	65
**Z2**	3	81	3	13
**Z3**	2	1	96	1
**Z4**	5	63	9	23
**Z5**	4	1	94	1
**Z6**	18	1	79	2

Atomic %

	C	Ga	Ag	In
**Z1**	10	3	30	57
**Z2**	15	76	2	7
**Z3**	14	2	83	0
**Z4**	26	56	5	13
**Z5**	25	2	72	1
**Z6**	66	1	33	1

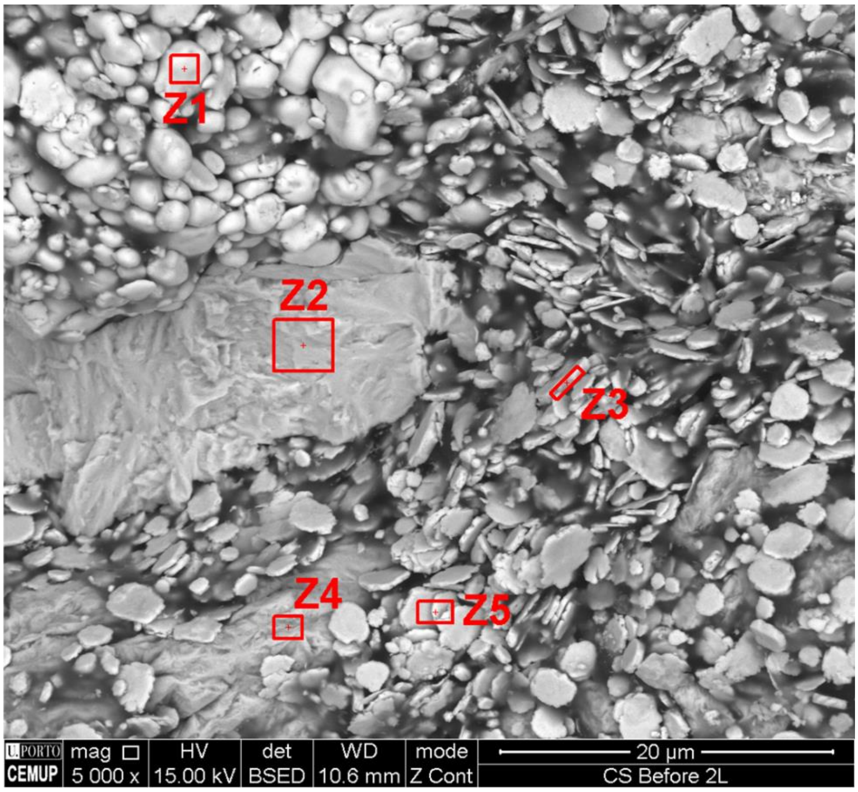

Supplementary Figure 7. **Scanning Electron Microscopy and energy dispersive X-ray spectroscopy analysis.** SEM and EDS analysis of the cross section of a sample shows creation of $AgIn_2$ microparticles (Z1), as well as Ag flakes (Z3,Z5), and Gallium Indium microdroplets (Z2,Z4)

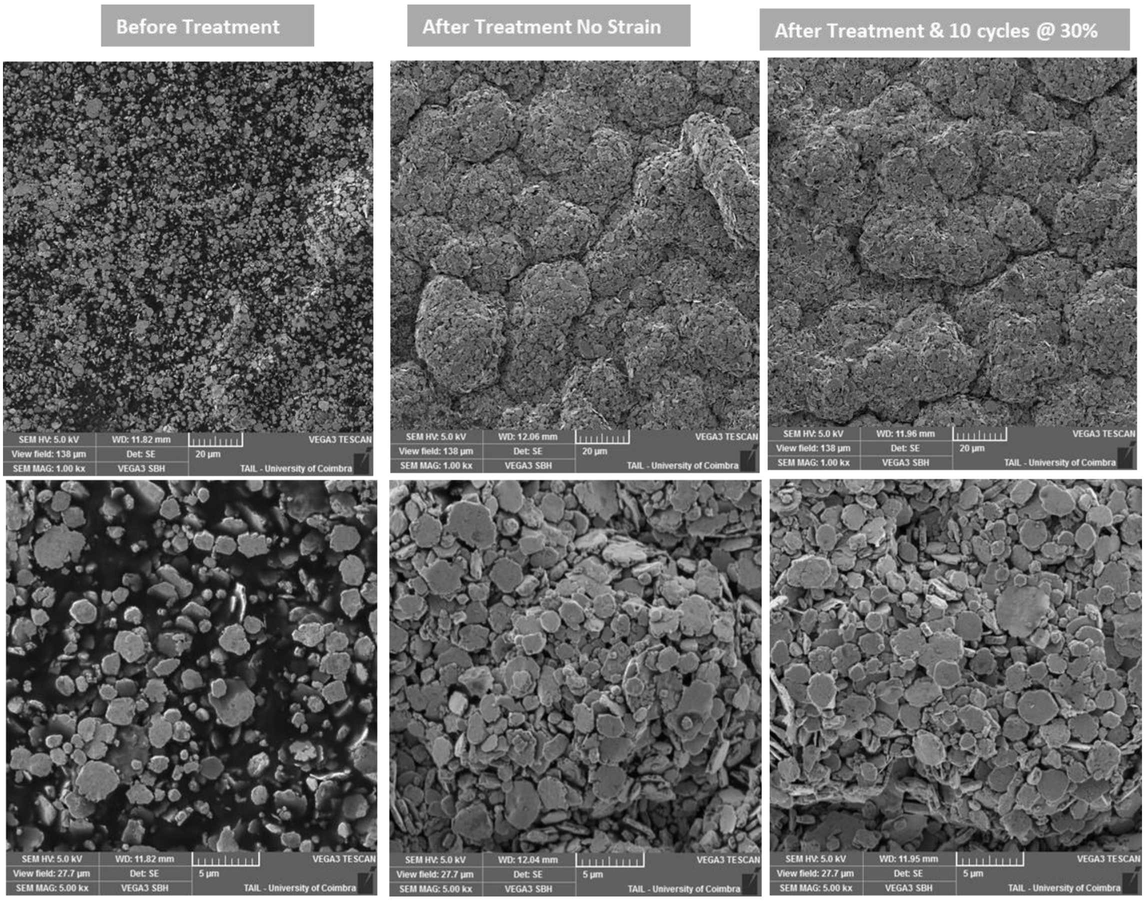

Supplementary Figure 8. **Scanning Electron Microscopy of sprayed ink.** SEM microscopy images of a sample of the ink spray coated over a piece of textile before treatment, after treatment, and after 10 cycles of 30% strain.

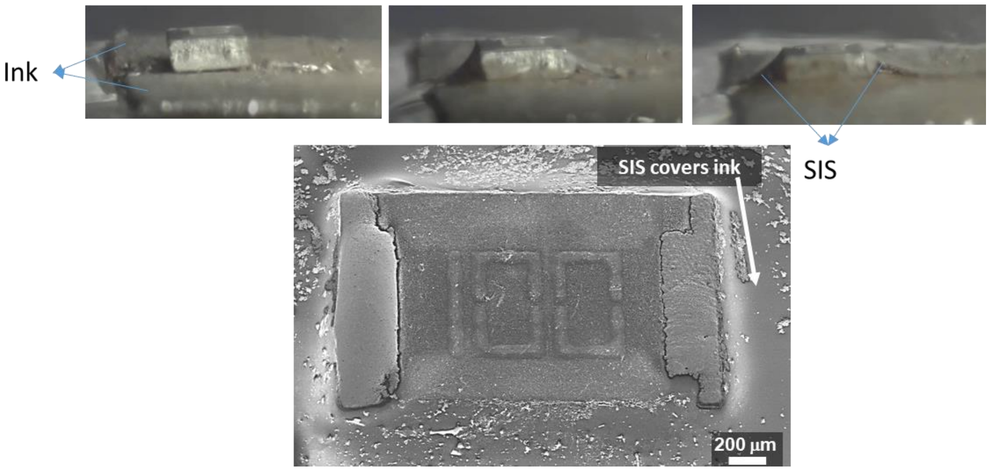

Supplementary Figure 9. **Vapor treatment of a circuit containing a chip resistor.** Top: Side view of a microchip before, during, and after the Pol-Gel process, which demonstrates the chip and ink penetration into the substrate, and the SIS attachment to the walls of the chip due to

capillary action. Bottom: a SEM image of the package after the treatment, which shows the self-coated trace.

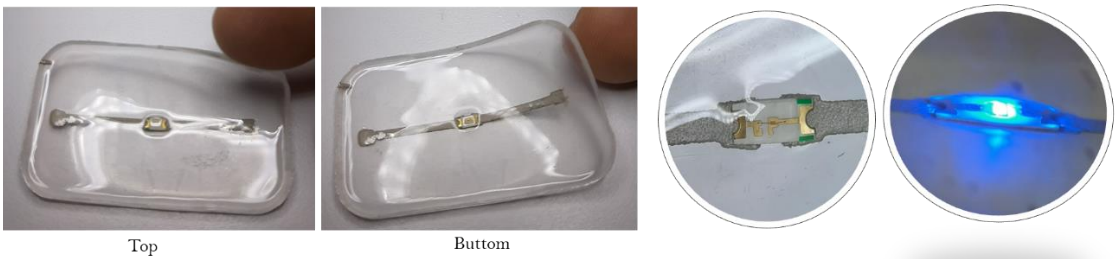

Supplementary Figure 10. **Fully coated sample**. The microchip is as well coated using the POL-GEL process. To do so, a 2mm thick substrate, was used. The printed trace drowned almost to the bottom of the substrate, but did not expose.

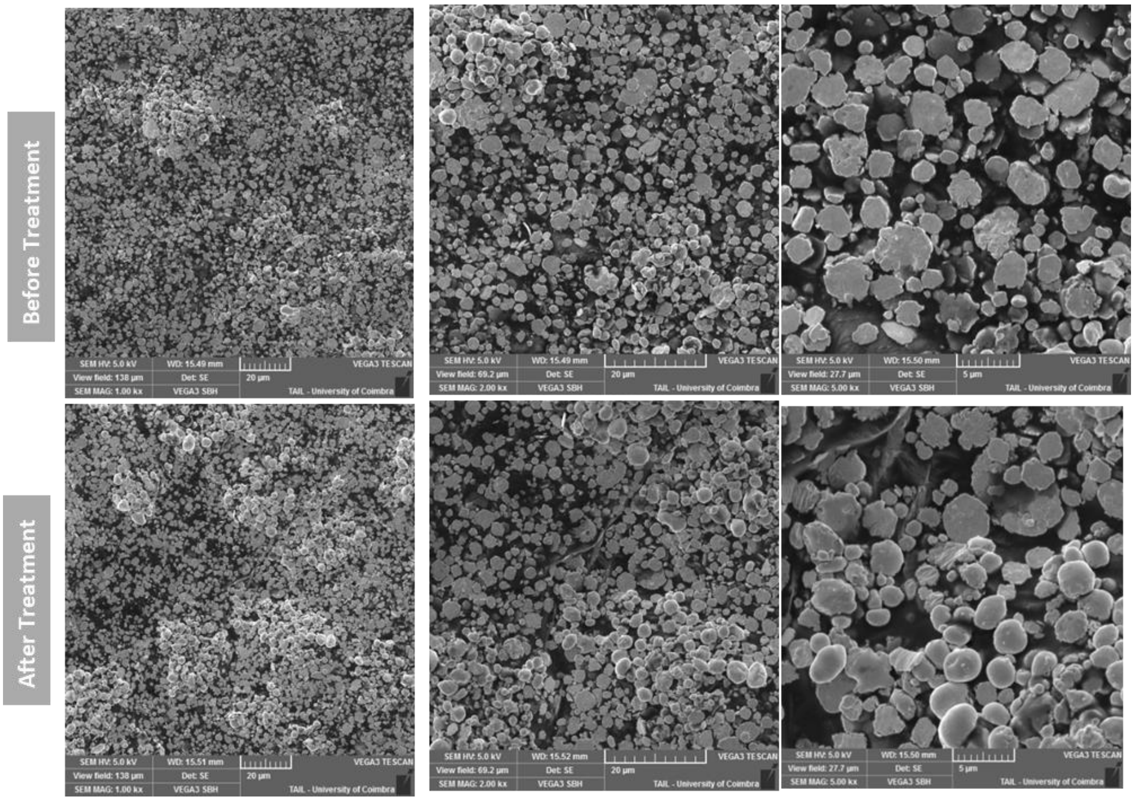

Supplementary Figure 11. **Scanning Electron Microscopy from a printed trace sample.** Additional SE images of the printed trace before and after vapor exposure.

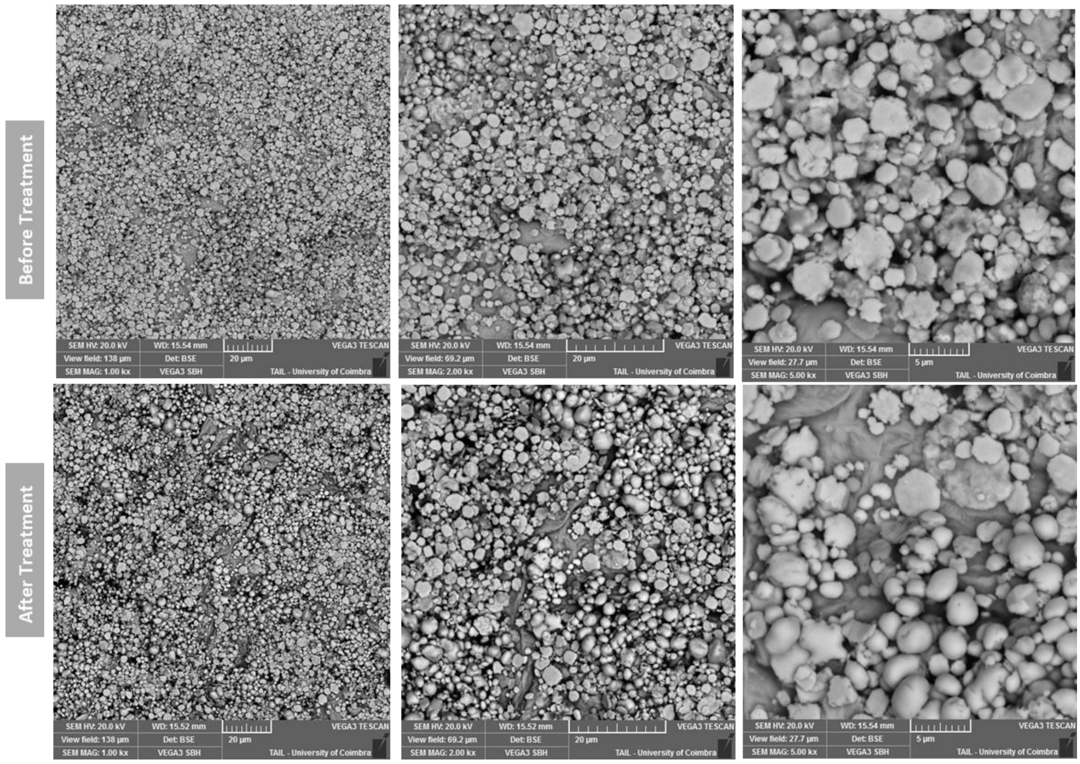

Supplementary Figure 12. **Energy dispersive X-ray spectroscopy analysis from a printed sample.** Additional BSE images of the printed trace before and after vapor exposure.

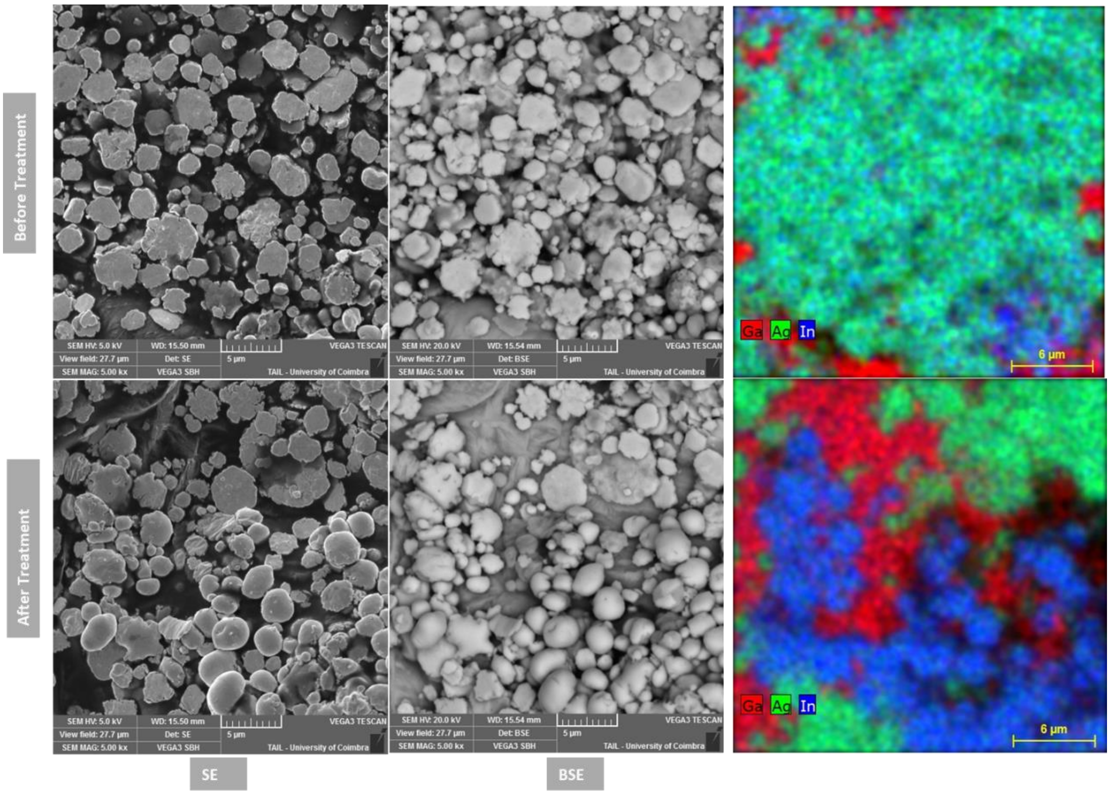

Supplementary Figure 13. **Compares SE, and BSE images, as well as EDS analysis.** The difference between the SE, and the BSE images demonstrate that before the treatment mostly Ag flakes are present at the top layer, and after the treatment, in addition to the flakes, also $AgIn_2$ is present at the surface of the sample. EDS analysis of the samples before and after treatment shows increased amount of Indium at the top surface. Before treatment the covering surface was mostly covered by Ag. Also from other SEM images after the treatment more $AgIn_2$ particles can be seen on the surface. This doesn't mean necessarily that there are more $AgIn_2$ formed, but that more of these particles are present at the top layer.

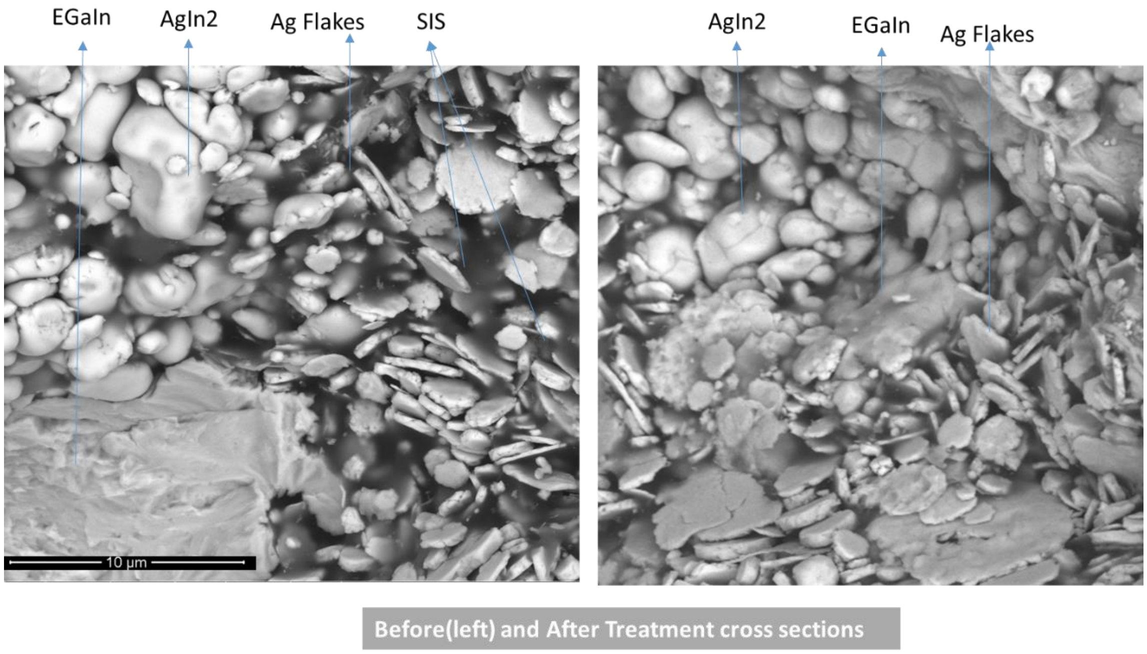

Supplementary Figure 14. **Additional cross section image of the sample.** Cross section prior to and after the Pol-Gel and subsequent Gel-Pol transition, where it is observable that after the process, the microstructure is considerably more compact.

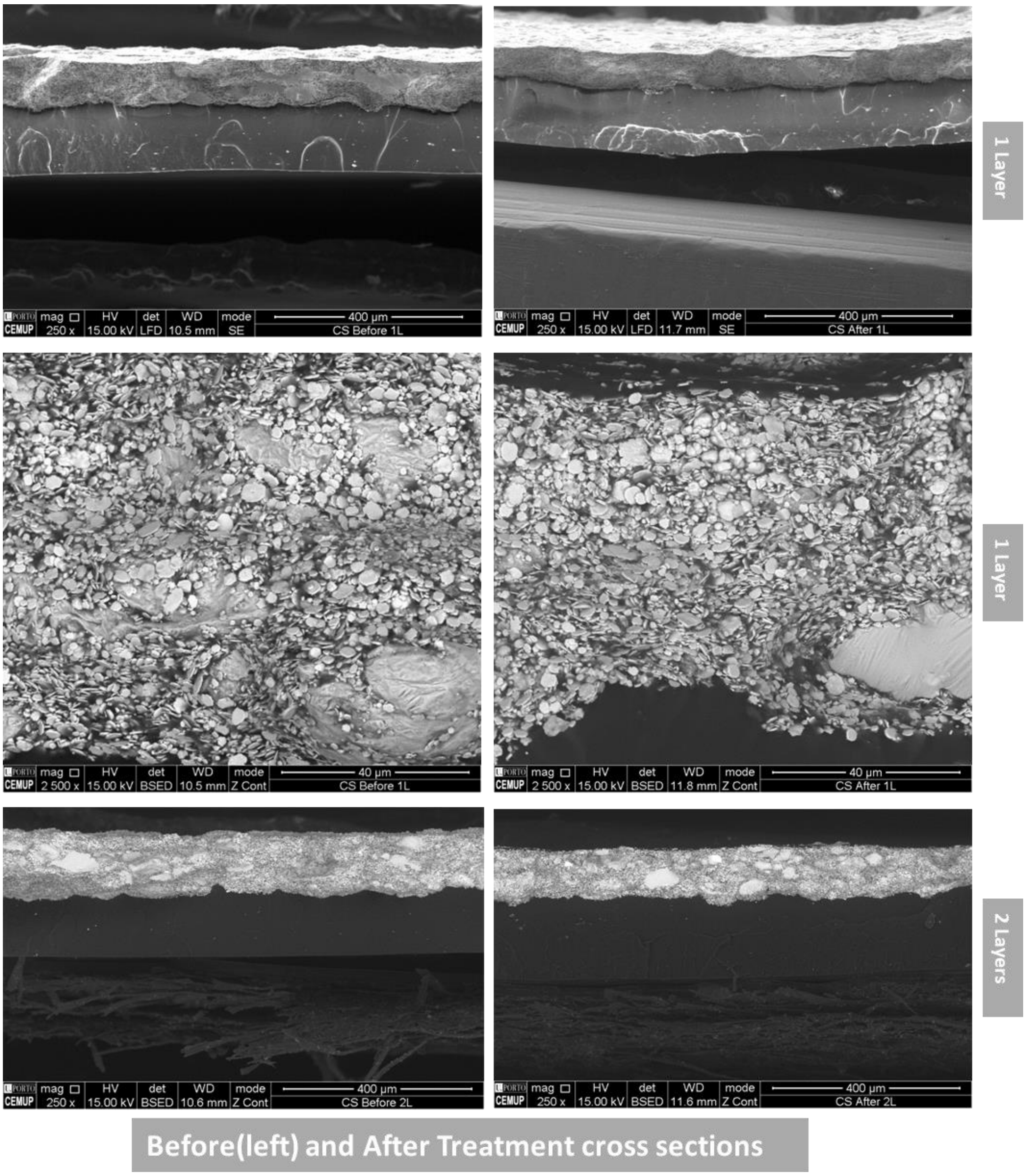

Supplementary Figure 15. **Additional SEM cross section**. Single and double layer printed trace, before and after the vapor exposure, where it is visible the reduction in the thickness of the sample.

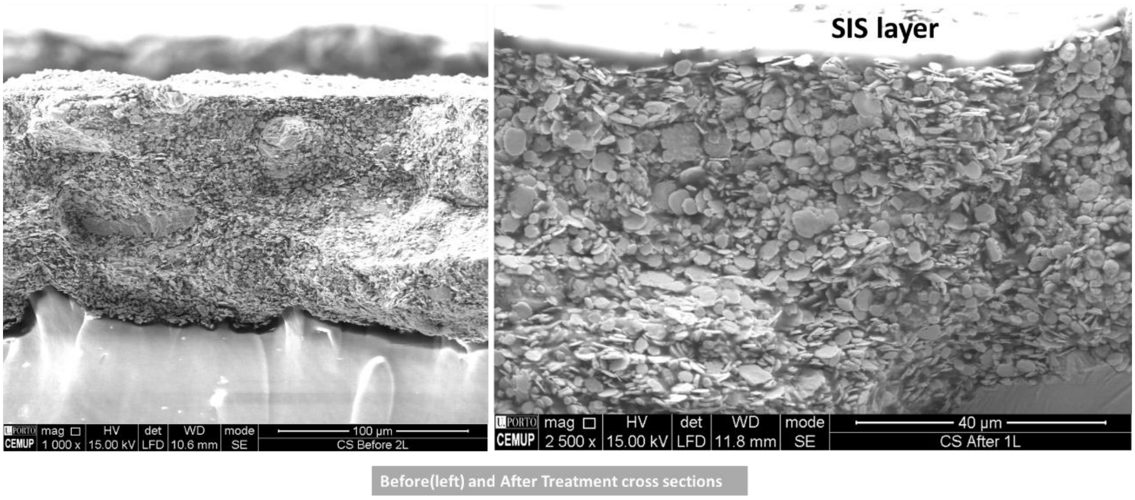

Supplementary Figure 16. **A high magnification SEM image.** Printed trace cross section, where the SIS coating over the printed trace is visible.

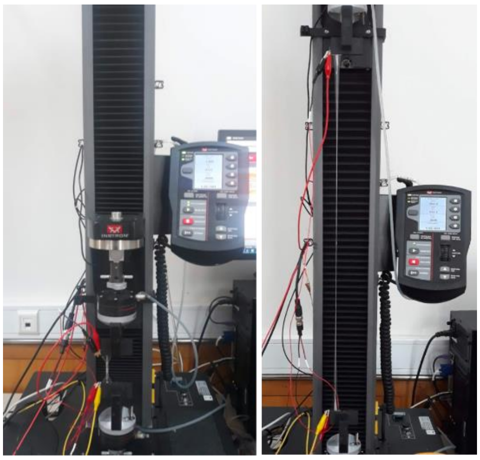

Supplementary Figure 17. **Strain test setup.** An example of a sample at 0%, and ~900% strain.

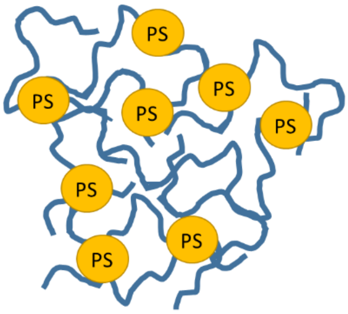

Supplementary Figure 18. **Chemical Structure of Styrenic Block Copolymers.** Polystyrene domains act as physical cross-linkers for isoprene chains.

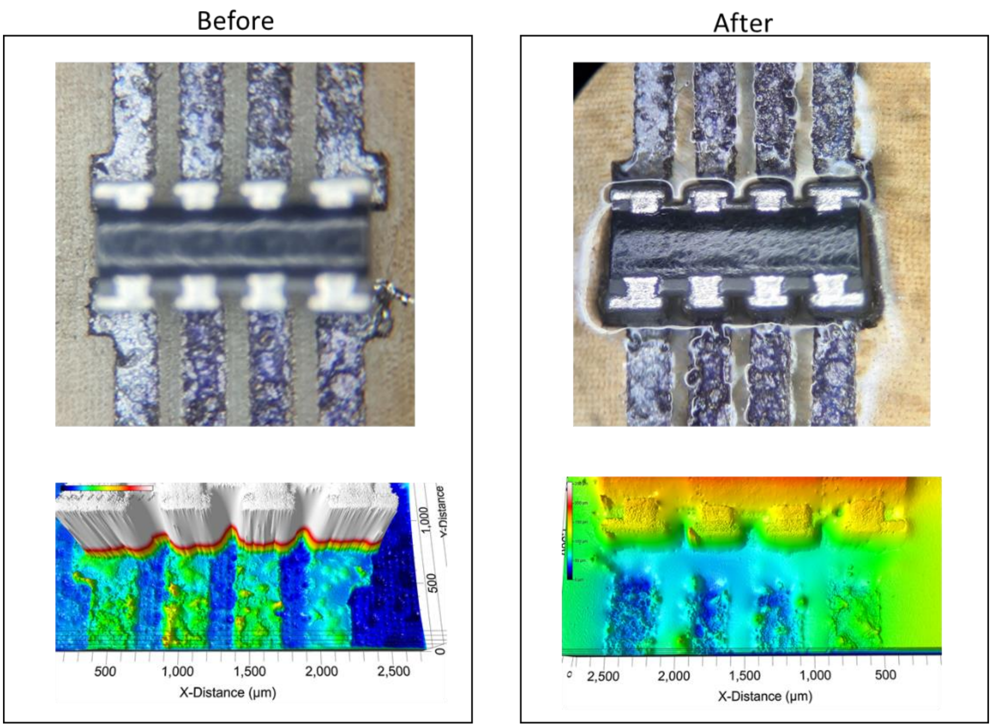

Supplementary Figure 19. **Vapor treatment of a circuit with a small pitch microchip.** A small resistor array chip, with 200µm spacing, before and after toluene treatment. No short circuits occur.
