# Supplementary material for: Reversible polymer-gel transition for ultra-stretchable chip-integrated circuits through self-soldering and self-coating and self-healing
Source: Nat Commun. 2021 Aug 3;12:4666. doi: 10.1038/s41467-021-25008-5 (PMC8333313; doi:10.1038/s41467-021-25008-5)
Supplement: Supplementary file 3 — Description of Additional Supplementary Files [file 41467_2021_25008_MOESM3_ESM.docx]

Supplementary Video 1. Shows the inside chamber footage of the Pol-Gel process for self-soldering and self-coating (Side view).

Supplementary Video 2. Shows the inside chamber footage of the Pol-Gel process for self-soldering and self-coating (Top view).

Supplementary Video 3. Shows the inside chamber footage of the Pol-Gel process for self-soldering and self-coating (Isometric view).

Supplementary Video 4. Shows the inside chamber footage of the Pol-Gel process for self-coating of the printed trace.

Supplementary Video 5. Shows an example of the cycle test of a dogbone with 4 LEDs.

Supplementary Video 6. Shows the resilience of the circuit after vapor exposure, when cutting it with a sharp knife.

Supplementary Video 7. Shows the inside chamber footage of the Pol-Gel process for self-healing of a cutted circuit, and the same sample under strain.

Supplementary Video 8. Shows various demo circuits produced by this technique, including 1. a LED display with 60 packages (LEDs and Resistors), 2. A wireless temperature and humidity monitoring patch with integrated Bluetooth, Microcontroller, Battery, Temperature and humidity sensor, and other packages, which sends the data wirelessly to a mobile phone application, and 3. A e-skin temperature measurement patch with LED display.
